# The Editor's Letter-Box

**Published:** 1914-05-02

**Authors:** Malcolm Stewart

**Affiliations:** Carlisle


*To the Editor of* The Hospital.

Sir,—From my point of view medicine as a profession had for some years previous to the advent of the panel been allowed to deteriorate to such an extent that a mark of distinction at present between panel and non-panel practitioners appears to me to be a matter of little consequence.—I am, etc.,

Malcolm Stewart, L.R.C.P., L.R.C.S.

Carlisle, April 26, 1914.

